# amplimap: a versatile tool to process and analyze targeted NGS data

**DOI:** 10.1093/bioinformatics/btz582

**Published:** 2019-07-26

**Authors:** Nils Koelling, Marie Bernkopf, Eduardo Calpena, Geoffrey J Maher, Kerry A Miller, Hannah K Ralph, Anne Goriely, Andrew O M Wilkie

**Affiliations:** 1 Clinical Genetics Group, MRC Weatherall Institute of Molecular Medicine; 2 Nuffield Division of Clinical Laboratory Sciences, Radcliffe Department of Medicine, University of Oxford, Oxford, UK

## Abstract

**Summary:**

amplimap is a command-line tool to automate the processing and analysis of data from targeted next-generation sequencing experiments with PCR-based amplicons or capture-based enrichment systems. From raw sequencing reads, amplimap generates output such as read alignments, annotated variant calls, target coverage statistics and variant allele counts and frequencies for each target base pair. In addition to its focus on user-friendliness and reproducibility, amplimap supports advanced features such as consensus base calling for read families based on unique molecular identifiers and filtering false positive variant calls caused by amplification of off-target loci.

**Availability and implementation:**

amplimap is available as a free Python package under the open-source Apache 2.0 License. Documentation, source code and installation instructions are available at https://github.com/koelling/amplimap.

## 1 Introduction

Targeted next-generation sequencing (NGS), for example from PCR-generated amplicons or capture-based methods, is widely used for screening of candidate disease genes in patient cohorts (Fenwick *et al.*, 2016) or for quantification of variant allele frequencies (VAFs) to detect allele-specific expression or mosaic mutations (Bernkopf *et al.*, 2017; Reijnders *et al.*, 2018).

Recently, targeted NGS techniques have also been extended to redundantly sequence the same original molecule of DNA multiple times to achieve very low error rates (Salk *et al.*, 2018). This enables the detection of somatic, sub-clonal mutations from cancer samples or mosaicism down to low levels (Acuna-Hidalgo *et al.*, 2017; Maher *et al.*, 2018). These high-fidelity protocols typically rely on the inclusion of unique molecular identifier (UMI) sequences, for example with single-molecule molecular inversion probes (smMIPs, Hiatt *et al.*, 2013).

However, significant computational work needs to be carried out to translate the raw sequencing reads generated by these protocols into interpretable genomic data, such as variant calls or VAFs. In practice, the processing and analysis of targeted NGS data often involves custom scripts, written specifically for the experimental design and dataset. Thus, each new analysis requires a significant amount of hands-on work from computational specialists and may be difficult to reproduce or repeat later.

**Fig. 1.**
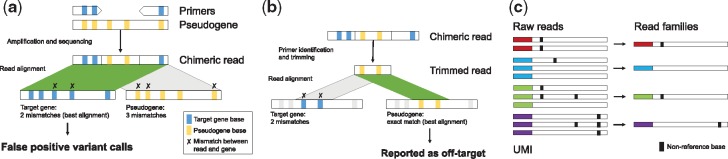
Illustration of custom features in amplimap. (**a**) False positive variants due to off-target events: Amplification of pseudogenes may generate chimeric reads that match the target gene better than the pseudogene, resulting in misalignment (left alignment, shown in green/dark grey) and pseudogene-specific bases being called as variants. (**b**) Trimming primers before alignment helps detect chimeric reads generated by off-target events: The trimmed read aligns to the pseudogene (right alignment, shown in green/dark grey), avoiding a false positive variant call. (**c**) Consensus calls are determined within each read family and filtered with user-defined stringency thresholds, resulting in more accurate allele counts. (Color version of this figure is available at *Bioinformatics* online.)

Furthermore, a common challenge is the unintended amplification of highly homologous loci, such as pseudogenes (Claes and De Leeneer, 2014). These loci may be amplified when primers inadvertently hybridize to highly homologous regions, creating chimeric reads that may lead to false variant calls (Fig. 1a). Currently, such false positives are often only identified through manual comparison to pseudogene sequences.

Here, we describe amplimap, a versatile tool that solves these challenges and enables the efficient processing and analysis of data from targeted NGS experiments.

## 2 Features and implementation

amplimap was developed in close collaboration with experimental scientists to ensure maximum user-friendliness and wide applicability. Processing a dataset only requires running a single command. Analyses can be customized in a variety of ways to fit the exact experiment performed. Input files are automatically checked for problems and tutorials are provided to act as a blueprint for common experiments. Results are provided as tables in CSV format, which can easily be loaded into Python, R or Excel for further analysis.

The main component of amplimap is the amplimap command-line executable, which has been tested with Python 3.5+ on current versions of Linux, MacOS and Windows (through the Windows Subsystem for Linux). Internally, amplimap uses the Snakemake work flow management package (Köster and Rahmann, 2012) to automate the efficient execution of external software as well as custom code. An overview of the pipeline and its input and output files is available from https://amplimap.readthedocs.io.

### 2.1 Annotated variant table and target coverage data

amplimap can create an aggregate table of all germline variants in all samples, including annotation such as the affected gene, the predicted deleteriousness of the variant and its frequency in reference populations. Additional target coverage tables give an overview of how thoroughly each target was sequenced in each sample.

### 2.2 Primer trimming and detection of off-target events

Primer sequences can be detected and trimmed by amplimap before alignment. This helps remove false positive variant calls caused by off-target amplification (Fig. 1b). The locations of off-target alignments are reported to allow further investigation. In addition, primer trimming also corrects skewed VAFs in regions where primers overlap another amplicon.

### 2.3 Read family consensus calls

To enable high-fidelity VAF quantification, amplimap can group reads based on identical UMIs (e.g. from smMIPs). When calculating allele counts and fractions, amplimap uses these read families instead of the individual reads to obtain more accurate, error-corrected consensus calls (Fig. 1c). The stringency of the consensus calls can be adjusted by specifying the minimum number of reads required to form a read family as well as the minimum fraction of reads supporting a consensus call. amplimap also calculates metrics such as the number of reads per family to provide quality control information and support future experimental design.

### 2.4 Tutorials

Tutorials are available from https://amplimap.readthedocs.io showing how to apply amplimap in three different contexts: variant calling in a patient panel, quantification of allele-specific expression and the identification of low-frequency somatic mutations with UMI-tagged smMIPs.
