# amplimap: a versatile tool to process and analyze targeted NGS data

**DOI:** 10.1093/bioinformatics/btz905

**Published:** 2020-02-26

**Authors:** Nils Koelling, Marie Bernkopf, Eduardo Calpena, Geoffrey J Maher, Kerry A Miller, Hannah K Ralph, Anne Goriely, Andrew O M Wilkie

*Bioinformatics* (2019) doi: 10.1093/bioinformatics/btz582, 35, 5349–5350

In the above article the following funding statement was initially omitted:

This work was supported by the Wellcome [grant numbers 102731 to A.O.M.W. and 105361 to H.K.R.], National Institute for Health Research (NIHR) Oxford Biomedical Research Centre (A.G. and A.O.M.W.) and Newlife (A.G. and A.O.M.W.).

